# Extended-wavelength diffuse reflectance spectroscopy dataset of animal tissues for bone-related biomedical applications

**DOI:** 10.1038/s41597-024-02972-3

**Published:** 2024-01-26

**Authors:** Celina L. Li, Carl J. Fisher, Katarzyna Komolibus, Huihui Lu, Ray Burke, Andrea Visentin, Stefan Andersson-Engels

**Affiliations:** 1 Biophotonics@Tyndall, IPIC, Tyndall National Institute, Lee Maltings, Dyke Parade, Cork, Ireland; 2 Insight Centre for Data Analytics, School of Computer Science and Information Technology, University College Cork, Cork, Ireland; 3 Department of Physics, University College Cork, Cork, Ireland

**Keywords:** Optical spectroscopy, Diagnostic markers

## Abstract

Diffuse reflectance spectroscopy (DRS) has been extensively studied in both preclinical and clinical settings for multiple applications, notably as a minimally invasive diagnostic tool for tissue identification and disease delineation. In this study, extended-wavelength DRS (EWDRS) measurements of *ex vivo* tissues ranging from ultraviolet through visible to the short-wave infrared region (355–1919 nm) are presented in two datasets. The first dataset contains labelled EWDRS measurements collected from bone cement samples and ovine specimens including 10 tissue types commonly encountered in orthopedic surgeries for data curation purposes. The other dataset includes labelled EWDRS measurements of primarily bone structures at different depths during stepwise drilling into intact porcine skulls until plunging into the cranial cavity. The raw data with code for pre-processing and calibration is publicly available for reuse on figshare. The datasets can be utilized not only for exploratory purposes in machine learning model construction, but also for knowledge discovery in the orthopedic domain to identify important features for surgical guidance, extract physiological parameters and provide diagnostic insights.

## Background & Summary

Orthopedic and neurosurgical specialties encompass the majority of bone-related surgical interventions to treat musculoskeletal or neurological conditions, which routinely require the surgeon to operate on both hard and soft tissues while avoiding damage to the surrounding vital organs including brain, spinal cord and major peripheral nerves. General orthopedic surgeries, such as total knee arthroplasty, and spine surgery, such as laminectomy, represented two of the three most frequently performed procedures in the United States in 2012^1^, mainly owing to population ageing. By 2018, spine fusion, knee and hip arthroplasty became the top three most costly principal operating room (OR) procedures, constituting approximately 24% of the top 20 OR procedures^2^. The incidence rate projection has also demonstrated a significant uptrend in the number of surgical cases to 2060, indicating an increase by 560% for primary total joint arthroplasty^3,4^ and 270% for posterior spinal fusion in patients aged >75 years^5^. In response to the growing demand, the number of orthopedic and neurological surgeons increased by 11% and 9%, respectively, from 2012 to 2017^6^.

Despite the increase in demand of orthopedic and neuro- surgery, reluctance of drilling is frequently witnessed with early-career practitioners due to insufficient experience and fear of excessive perforation causing severe damage^7^. Studies have found that surgical learning curves to these high-volume procedures can be significantly steep, requiring a substantial amount of time, effort and practice before mastery is attained^8–10^. The need for extensive training or new guidance devices thus becomes imperative to improve operative efficiency, patient outcome and safety. The risk of plunging and breaching into critical structures can be greatly reduced by implementing safety measures into the surgical tools so that either the mechanical processes can be automatically terminated in a timely manner or the exact drilling speed and depth can be monitored in real time. Several approaches have been investigated including optical methods^11,12^, surgical techniques^13–15^ and mechanical tools^16,17^. It has, however, remained technically challenging for surgeons to navigate through bone-related surgical procedures because of limited visibility and a restricted field of view, though various intraoperative imaging techniques can be utilized for enhanced visualization and accuracy combined with computer-assisted technologies^18–20^.

Diffuse reflectance spectroscopy (DRS) is an established technique routinely used for measuring electromagnetic radiation reflected off sample surfaces as a function of wavelength^21^. The utilization of DRS in preclinical and clinical settings has been extensively studied for the purpose of diagnostic guidance in a wide range of applications, notably as a promising aid to cancer delineation in breast^22^, liver^23^ and gastrointestinal tract^24^. With relatively low-cost instrumentation and easy maneuverability, this spectroscopy method can offer non-contact to minimally invasive, usually point-based interrogation of tissues of interest based on their wavelength dependent absorption and scattering properties. Furthermore, machine learning (ML) algorithms are often seen deployed on DRS datasets for tissue classification and parameter extraction due to the information-rich nature^25–27^. ML can facilitate selection of the most important spectral features and help evaluate the diagnostic power of the technique.

Recently our and other groups introduced and discussed perspectives on optical integration to guide bone-related surgeries, of which the proposed techniques could potentially provide more effective surgical approaches with less side effects^28–30^. Along these lines we have demonstrated the potential of DRS for tissue differentiation in orthopedic procedures^31–33^, and subsequently described four distinct feature selection frameworks formulated based on DRS measurements^34^. The aim of this specific publication was to discover a reduced number of wavelength features with sufficient discriminative power for tissue differentiation, thus decreasing instrumentation complexity of optical systems for clinical translation.

The publications in this field are, however, so far scarce and there is a need for more open-source data. This study was thereby designed to facilitate data re-utilization and the development of ML models for data mining tasks in bone-related biomedical applications. Two datasets are included with a technical analysis as validity check. Both datasets fulfill the pre-requisites for ML and deep learning (DL) techniques by collecting sufficiently large data libraries with labels from multiple *ex vivo* tissue specimens.

**Fig. 1:**
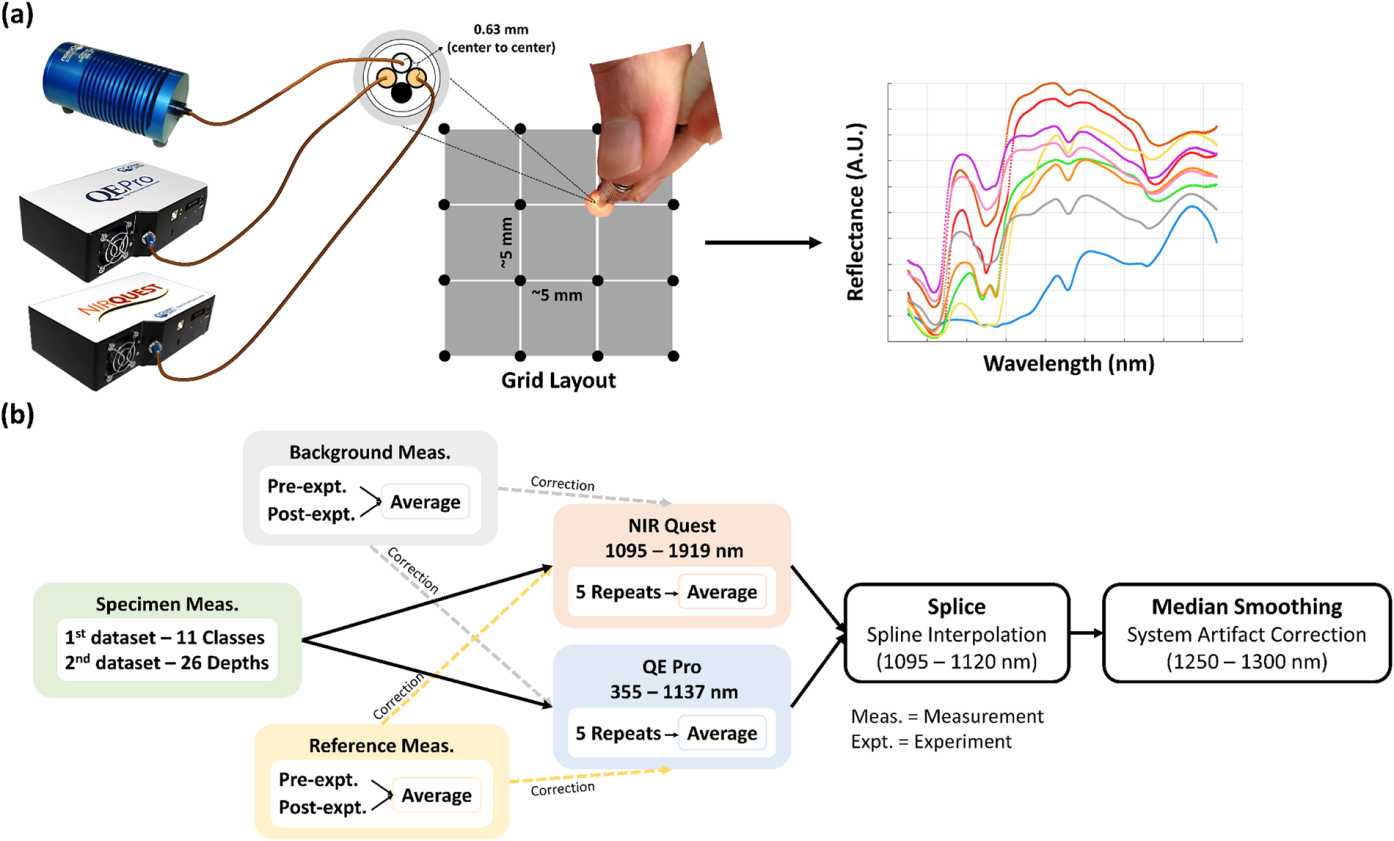
EWDRS dataset collection and pre-processing workflow. (**a**) Modified from the previous publication^34^. Experimental setup included two commercialized spectrometers and a tungsten-halogen broadband light source. The source-detector separations were 0.63 mm from center to center. The locations of measurements followed a grid layout of approximately 5 mm apart for all specimens. (**b**) Data pre-processing consisted of background correction, reference calibration, spectral splicing using spline interpolation and median smoothing.

In the data descriptor, we explain in detail the experimental collection of two extended-wavelength DRS (EWDRS) datasets. EWDRS measurements ranging from 355 to 1919 nm were acquired from *ex vivo* specimens sourced from a local butcher shop in Cork, Ireland. All measurements were conducted employing a broadband light source for illumination with two commercialized spectrometers for simultaneous signal detection. As shown in Fig. 1a measurements were acquired in a grid pattern of ~5 mm apart, while Fig. 1b summarizes the workflow of EWDRS data pre-processing.

**Fig. 2:**
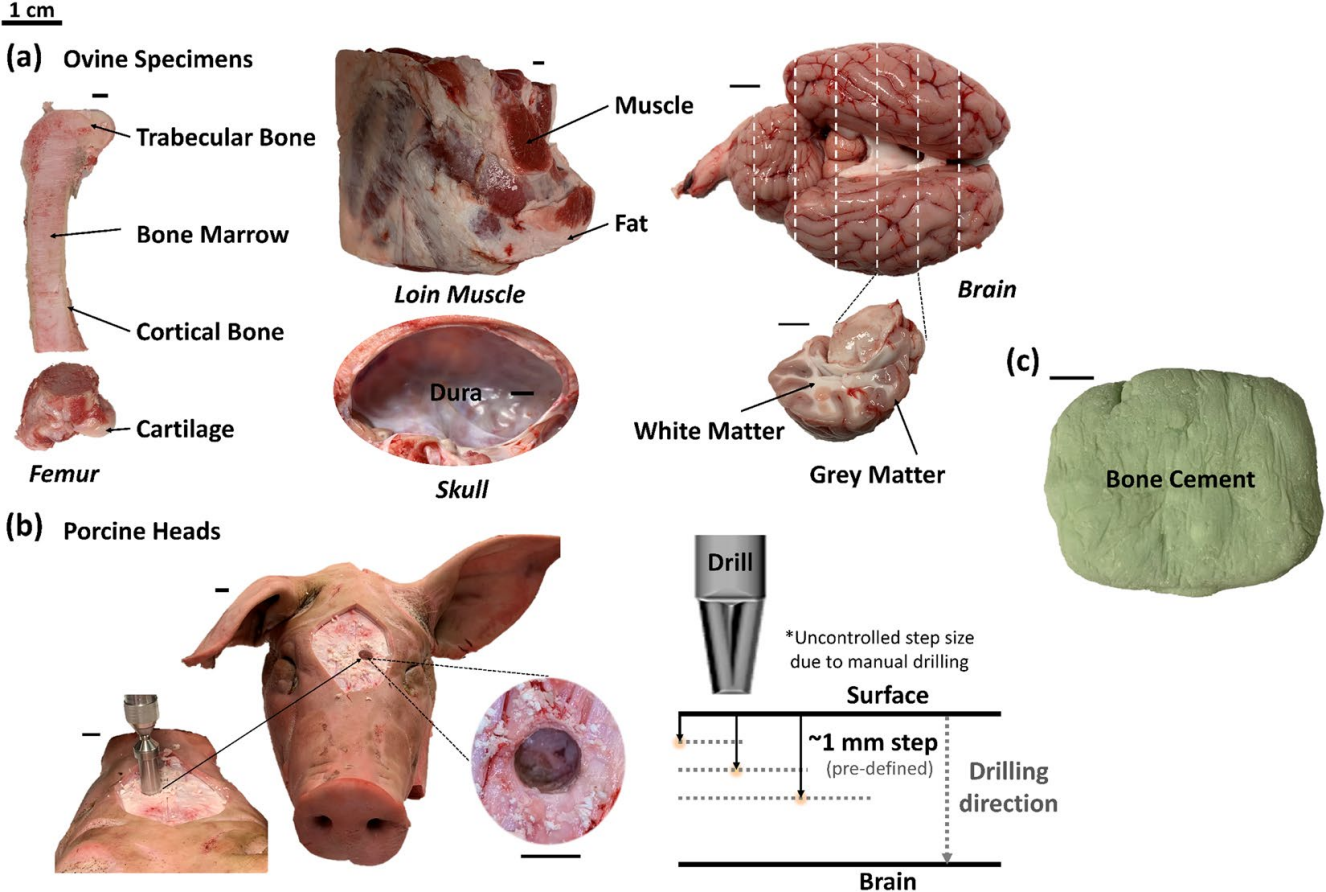
Example *ex vivo* specimens used for EWDRS dataset creation. (**a**) Ovine specimens included bone marrow, cartilage, cortical bone, trabecular bone (femur), dura, fat, muscle, grey and white matter (brain). Whole brain tissues were extracted from ovine cranial cavities and sectioned along the coronal plane to expose grey and white matter. (**b**) Stepwise drilling was performed manually on porcine heads into the cranial cavity from the surface. The step size was pre-defined to be 1 mm but was uncontrolled during drilling due to manual handling. EWDRS measurements were taken at each stop. (**c**) Adapted from the previous publication^34^. Bone cement samples were cured and hand-molded into blocks. The black scale bars represent 1 cm.

The first dataset (Dataset 1) was created for general orthopedic applications and skull-based procedures, which included tissue types commonly encountered during the operation. This dataset was included for data curation purposes^34^. As shown in Fig. 2a, Dataset 1 comprised data from 10 biological tissue types of ovine specimens, which were bone marrow, cartilage, cortical bone, endosteum, fat, muscle, trabecular bone, dura, grey and white matter. One non-biological specimen, namely bone cement, was also included in this dataset (Fig. 2c) since the targeted clinical application was optical guidance of bone cement removal in total hip arthroplasty. Only Part II of Dataset 1 is included in this data descriptor, as Part I was previously published in our previous publication^34^ and shared on Zenodo^35^. Part II of Dataset 1 includes class labels for endosteum, fat, dura, grey and white matter. The second dataset (Dataset 2) was created for neurosurgical applications involving craniotomy, which included depth measurements of porcine skulls. This dataset consisted of EWDRS measurements of primarily cortical bone at different depths during stepwise drilling of porcine skulls until plunging into the cranial cavity (Fig. 2b).

The collection of the published data library was motivated by the need to identify important spectral features for surgical guidance and better comprehend the knowledge space of DRS relating to orthopedics and neurosurgeries. The exploration could set the groundwork to select adequate wavelengths for tissue differentiation, predict layer thickness of cranial bones and benchmark classification metrics as a reference. For reuse, we believe that the data is useful for translational optics research and can enable the possibility to develop suitable ML or DL models utilizing DRS measurements. It can also boost the deployment of medical DL approaches based on EWDRS through transfer learning, which has gained considerable attention recently in spectroscopy^36,37^. While there has been spectroscopy data published in the literature^38^, our data library not only encompasses a significantly broader spectral range covering the ultraviolet A, visible, near infrared and short-wave infrared (UVA/VIS/NIR/SWIR) windows, but also provides measurements of more biological tissue types in greater amounts.

## Methods

The following subsections describe the full experimental protocols of data collection and pre-processing expanded upon our previous work^34^, where readers can find the details for collecting Dataset 1 (Fig. 2a). All *ex vivo* measurements were approved by and in compliance with the regulations at Tyndall National Institute, Cork, Ireland.

### Specimen preparation and setup

The tissue sample preparation used to collect Dataset 1 (Fig. 2a) is described in detail previously^34^. Briefly, freshly refrigerated ovine tissues 2 days post slaughter from a local butcher was immediately measured upon delivery. The cross sectional opening of ovine skulls representing dura was approximately 9 × 5 cm on average. Brain containing grey and white matter was approximately 11 × 8 × 3 cm on average in the relaxed form, which was sectioned coronally into approximately 1-cm thick slabs. Dataset 2 included EWDRS measurements acquired from 5 porcine heads, of which the superficial layer of skin and connective tissue were shaved off to expose the skull (Fig. 2b). A commercial surgical drill (CD4, Stryker, USA) as previously reported^33^ with no optical enhancement was employed, which was manually handled for drilling to closely approximate a stepwise thickness of 1 mm. No a priori knowledge of skull thickness was obtained. The thickness of the cranial bone was variable depending on the initial drilling location. As a result, the exact drilling depth per stop was uncontrolled, leading to a different number of measurements recorded at each pre-defined depth level. For instance, no measurement was collected at 23-mm thickness of the skull.

### Extended-wavelength diffuse reflectance spectroscopy

The experimental system is illustrated in Fig. 1a and described in our previous work^34^, with the data pre-processing shown in Fig. 1b. The DRS system consisted of dual spectrometers, measuring the 355 to 1137 -nm range (QEPro-FL, QE Pro spectrometer, Ocean Insight B.V., Duiven, The Netherlands) with approximately 6.9 nm optical resolution and a spectral bin width of approximately 0.76 nm, and the 1095 to 1919 -nm range (NIRQUEST512-1.9, NIR Quest spectrometer, Ocean Insight B.V., Duiven, The Netherlands) with approximately 12 nm optical resolution and a spectral bin width of approximately 1.6 nm per channel, respectively. The specimens were illuminated by a tungsten-halogen light source (8.8 mW, 350–2400 nm, HL-2000-HP, Ocean Insight B.V., Duiven, The Netherlands) and probed for 2 s by a commercialized fiberoptic bundle (BF46LS01, 600-μm core, Low OH, Thorlabs, Munich, Germany) as shown in Fig. 1a. For data acquisition, 5 repeats were included for each spectrometer. During each repeat, 250 spectra and 1 spectrum were recorded by the QE Pro spectrometer and the NIR Quest spectrometer with an individual exposure time of 8 ms and 2 s, respectively, to achieve a good signal-to-noise ratio. All specimens except dura were prepared with at least a 1-cm thickness. Dura was prepared as a single layer membrane. The overlapped region was utilized for spectral splicing. It is recommended to trim off the spectral range beyond 1850 nm for further interpretive analysis due to increased noise level. One measurement is equivalent to one EWDRS spectrum. Specific details for the two datasets are summarized in Table 1.

**Table 1:** Summary of the EWDRS measurements included in Dataset 1 (left) and Dataset 2 (right).

	Dataset 1			Dataset 2	
	Spec. Type	No. of Spec.	No. of Meas.		
Part I	Bone Marrow	43	1000	**Total No. of Heads**	5
				**Total No. of Drilled Holes**	11
	Cartilage	55	1000	**Total No. of Meas**.	102
	Cortical Bone	48	1000	**Min. Thickness (mm)**	0 (brain)
	Muscle	31	1000	**Max. Thickness (mm)**	25 (surface)
	Trabecular Bone	47	1000		
	Bone Cement	3	215		
Part II	Endosteum	38	245		
	Fat	28	1000		
	Dura	9	215		
	Grey Matter	78	413		
	White Matter	92	436		
Total		472	7524		

Spec. = Specimen, Meas. = Measurements, No. = Number, Max. = Maximum, Min. = Minimum, Thickness = Distance away from the brain.

### Data Pre-processing

**Fig. 3:**
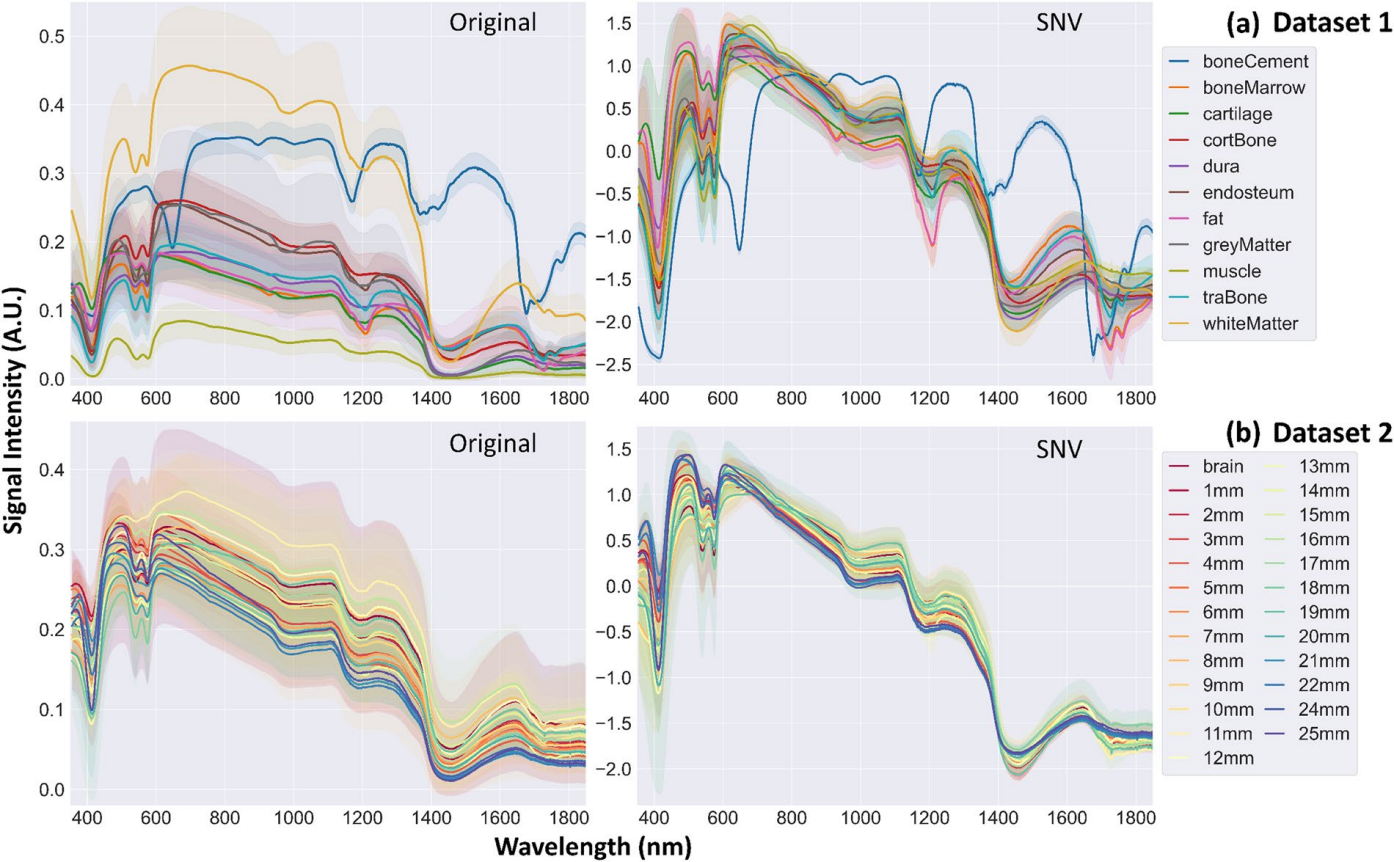
The averaged EWDRS spectra for all labels in (**a**) Dataset 1 and (**b**) Dataset 2 showing the original spectra (left) and the SNV transformed spectra (right). SDs are shown by shaded error bands. Spectral range beyond 1850 nm was trimmed off.

Data pre-processing was expanded upon our previous work^34^ with more details, which was implemented in both the Python Programming Language Version 3.9 (Python Software Foundation, https://www.python.org/) and MATLAB (R2020b, The MathWorks Inc., Natick, Massachusetts, USA, https://www.mathworks.com). All code scripts for data pre-processing described here can be found in Code Availability. Due to the dual spectrometer configuration, two spectra were generated. Splicing via spline interpolation was performed over a selected range between 1095 and 1120 nm of the overlapped region using 1D interpolation (MATLAB default function). Background correction and reference calibration were calculated by Eq. 1:

1 $$Calibrated\;EWDRS\;spectrum=\frac{{S}_{raw}-{S}_{bkgd}}{{S}_{ref}-{S}_{bkgd}}$$

where *S*_*raw*_ represents the averaged raw DRS spectrum over five repeats, *S*_*bkgd*_ represents the averaged DRS spectrum of the background pre and post the experiment (without illumination light), and *S*_*ref*_ represents the averaged DRS spectrum of the reflectance standard (FWS-99-01c, Avian Technologies LLC, New London, USA) pre and post the experiment (with illumination light). The spectral bin width over the spliced region was 0.23 nm. Class labels were recorded in the last column for both datasets. A systematic artifact from the spectrometer between 1250 and 1300 nm was corrected by a smoothing method using moving median with a window size of 2.2 (nm). Standard normal variate (SNV) transformation, a spectral scaling technique calculated by Eq. 2, was used as scattering correction to remove unrelated offsets:

2 $$SNV\;EWDRS\;spectrum=\frac{\left({S}_{cal}-\overline{{S}_{cal}}\right)}{SD\left({S}_{cal}\right)}$$

where *S*_*cal*_ represents the calibrated spectrum from Eq. 1, $\overline{{S}_{cal}}$ represents the mean of *S*_*cal*_ and *SD*(*S*_*cal*_) represents the standard deviation (SD) of *S*_*cal*_. Figure 3 illustrates the averaged spectra for all labels in the two datasets, excluding the spectral range from 1850 to 1919 nm.

## Data Records

Part II of Dataset 1 and the full Dataset 2 are publicly available in a figshare repository^39^. The pre-processed data is stored in CSV files with headers (wavelengths) in the first row and class labels (tissue types or thicknesses) in the last column. Both the original and the SNV transformed data are provided in separated CSV files. There are 7524 and 102 EWDRS measurements in the full Dataset 1 and Dataset 2, respectively, and 1531 wavelength channels in total (trimmed beyond 1850 nm). Since Part I of Dataset 1 was analyzed previously and shared on Zenodo^35^, Dataset 1 in the figshare repository contains only Part II. The full Dataset 1 in one CSV file for easy access and formatting is available upon request. The class label column of Dataset 2 is formatted in both strings and numerical values in separate files for easier utilization with classification and regression tasks, respectively. Raw data with background and reference measurements is provided in both CSV files and MAT files. Metadata and licensing information are stored in the corresponding dataset folders.

The Dataset 1 folder “EWDRS_dataset_ortho” contains:

EWDRS_dataset_ortho/

- raw_data_archive/(Wavelength Range: 355–1919 nm)
  - 15 subfolders (e.g. Expt_Dat_01/) representing individual experimental sessions containing:
    - cal_proc_csv/ including calibrated and processed data in CSV.
    - cal_proc_mat/ including the same as above in MAT.
    - cal_proc_plot_norm/ including normalized spectral plots.
    - raw_csv/ including raw, background, reference data in CSV.
    - raw_mat/ including raw, background, reference data in MAT.
- all_EWDRS_meas_cal_proc_new.csv: the processed original dataset (Part II), 355–1850 nm.
- all_EWDRS_meas_cal_proc_new_SNV.csv: the SNV transformed dataset of above.
- number_of_samples_ortho.xlsx: detailed information on specimens for each experiment.
- LICENSE.txt: licensing information.
- README.txt: basic information of the dataset.

The Dataset 2 folder “EWDRS_dataset_drilling_depth” contains:

EWDRS_dataset_drilling_depth/

- cal_proc_csv/ including 26 CSV files of calibrated and processed data for individual class labels, 355–1919 nm.
- cal_proc_mat/ including the same as above in MAT.
- drilling_cal_proc_all.csv: the fully processed original dataset with class labels in strings, 355–1850 nm.
- drilling_cal_proc_all_snv.csv: the SNV transformed dataset of above.
- drilling_cal_proc_all_regr.csv: the fully processed original dataset with class labels in numerical values, 355–1850 nm.
- drilling_cal_proc_all_snv_regr.csv: the SNV transformed dataset of above.
- LICENSE.txt: licensing information.
- README.txt: basic information of the dataset.

## Technical Validation

Technical validation and integrity were ensured by following rigorous experimental protocols and data pre-processing steps. Data was acquired under controlled environmental factors from *ex vivo* specimens that were prepared and handled consistently throughout all sessions. Tissue identification was confirmed by a medical resident. Background and reference measurements against the same reference standard were always collected pre and post each experimental session to account for both inter- and intra- day variations and offsets using the standard correction method (Eq. 1). The selected wavelength range spanning the UVA/VIS/NIR/SWIR windows was appropriate with the use of the illumination light source to provide more spectral information. No tissue damage was identified from the use of the light source.

Upon initial examination of the original spectral curves in Fig. 3, spectral features of typical biomarkers demonstrating absorption were detected, including oxy-hemoglobin near 414, 543 and 577 nm, deoxy-hemoglobin near 433 and 556 nm, as well as collagen, lipids and water near 1200, 1430 and 1750 nm^40^, respectively, which were comparable to the literature^41^. The magnitude of SDs was variable depending on the class label while the error bands followed the same trend as the averaged spectra. For Dataset 1, brain tissue demonstrated the most variations, where the maximum was found in grey matter showing a ± 0.11 SD at 695 nm with an original data value of 0.25. For Dataset 2, the maximum was also found in brain showing a ± 0.13 SD at 875 nm with an original data value of 0.28. The behavior broadly suggested that no presence of outlier was detected, and the degree of variability was primarily caused by intrinsic specimen heterogeneity. This was further verified by the observation that SDs diminished significantly after applying SNV transformation, especially for Dataset 1. Larger variations were indeed found near the spectral absorption features. The degree of SDs on the SNV-transformed Dataset 2 was marginally variable, as expected, because the data was mostly acquired from the same biological tissue type. Due to low noise level in the entire spectra, data smoothing was only applied to correct one systematic artifact instead of the whole range.

The two datasets encompass a sufficiently large amount of EWDRS measurements for use in ML and DL modeling. More data can be reproduced efficiently and reliably following the steps outlined here. Class imbalance can else be corrected by over-sampling techniques such as Synthetic Minority Over-sampling Technique (SMOTE) or under-sampling methods using K-means or nearest neighbors algorithms. As discussed in our previous manuscript^34^, technical limitations not only existed in the instrument setup since measurements were collected from two adjacent acquisition volumes, but also in the experimental environment because the reported data was not collected from an *in vivo* setting and thus became less adequate for simulating ongoing clinical scenarios. In addition, sample limitations were present in the *ex vivo* non-human specimens employed in the study. The findings might not be completely generalizable to clinical scenarios due to altered amounts of blood, lipid and water contents as well as the oxygenation states of hemoglobin and overall different tissue microenvironments, which would lead to changed optical properties and DRS measurements as compared to clinical trials^42^. Nevertheless, porcine models demonstrate gross similarities in optical properties and tissue heterogeneity with human subjects such as in brain and bone, and have been seen increasingly used in orthopedic research^42–45^. It is therefore believed that the present dataset can serve for reuse to facilitate development of clinical optical diagnostic and monitoring tools with artificial intelligence implementation.

## Data Availability

Example codes are publicly available in a Github repository at: https://github.com/Biophotonics-Tyndall/PUB-DataDescriptorCode.git. The repository contains one MATLAB script (DRS_cal_proc_example.m) demonstrating DRS data processing and systematic artifact correction, one Python function (calibDRS.py) for SNV transformation, and three MATLAB functions to namely calculate area-under-curve (AUC_DRS_fnc.m), splice two spectra via spline interpolation (DRS_splice_fnc.m) and calculate SNV transformation (snv_DRS_fnc.m) for DRS data.
